# Knowledge, Attitudes, and Practices of Antenatal Women Regarding Vaccination, and Determinants of Vaccination Acceptance: A Mixed-Methods Study

**DOI:** 10.3390/vaccines14100876

**Published:** 2026-10-02

**Authors:** Rajani Dube, Bellary Kuruba Manjunatha Goud, Yosra Ghassan Habra, Sarah Dawood, Yasmin Sharifian, Subhranshu Sekhar Kar, Mohammed Mahmood Ali, Omar Mohamed Alahmed, Hamad Easa Alshamsi, Swayam Siddha Kar

**Affiliations:** 1 Department of Obstetrics and Gynecology, RAK College of Medical Sciences, RAK Medical and Health Sciences University, Ras Al Khaimah P.O. Box 11172, United Arab Emirates; yosra0569@gmail.com (Y.G.H.); sndawood20@gmail.com (S.D.); yasminsh.ys98@gmail.com (Y.S.); mohammed.23901054@rakmhsu.ac.ae (M.M.A.); omar.23901109@rakmhsu.ac.ae (O.M.A.); hamad.23901126@rakmhsu.ac.ae (H.E.A.); 2 Department of Biochemistry, RAK College of Medical Sciences, RAK Medical and Health Sciences University, Ras Al Khaimah P.O. Box 11172, United Arab Emirates; manjunatha@rakmhsu.ac.ae; 3 Department of Pediatrics, RAK College of Medical Sciences, RAK Medical and Health Sciences University, Ras Al Khaimah P.O. Box 11172, United Arab Emirates; subhranshu.kar@rakmhsu.ac.ae; 4 Department of Obstetrics and Gynecology, School of Medicine, University of Lancashire, Preston Campus, Preston PR1 7BH, UK; swaekar35@gmail.com

**Keywords:** antenatal vaccination, knowledge about vaccination service, attitude and practice of vaccination, acceptance of vaccine, vaccine hesitancy

## Abstract

**Background/Objectives**: Vaccination is the most important way to prevent infection-related morbidity and mortality, but delays in the acceptance or refusal of vaccines despite the availability of vaccination services can adversely affect health outcomes. **Methods**: This is a cross-sectional congruent parallel mixed-methods study exploring vaccine-related knowledge, attitudes and practices and factors associated with vaccine acceptance or hesitancy. A validated questionnaire was used to collect responses from pregnant women. **Results**: A total of 326 women participated in the study. The majority of women were over 30 years old, educated, had higher incomes, and had been pregnant before. Of the participants, 94.7% had adequate knowledge of vaccine services, 64% relied on more than one source of information, and the majority (89%) relied on information from doctors. In total, 93.5% held a positive attitude towards vaccination, and the majority accepted it. A total of 159 (48.7%) self-reported a moderate to strong vaccine hesitancy (VH). Qualitative analysis showed that those who had strong VH expressed mistrust, social media information, safety concerns regarding newer vaccines, and religious beliefs as influencing factors. Factors significantly affecting VH were education, household income, adequate knowledge, and appropriate attitude. Other factors were knowledge gaps, perceived necessity, distrust, and belief in natural immunity. Hesitancy positively correlated with being a homemaker and being parous, but the difference was not statistically significant. **Conclusions**: This study reveals a population with generally favorable vaccine attitudes and high vaccination intentions, alongside significant underlying hesitancy. Knowledge gaps exist, and healthcare professionals remain the most trusted information source. Addressing the identified barriers through evidence-based, culturally sensitive, and community-engaged strategies will be essential for maintaining high vaccination coverage and preventing future outbreaks.

## 1. Introduction

Vaccination is the most important public health intervention for preventing morbidity and mortality due to infectious diseases globally. It is generally accepted across populations. However, vaccine hesitancy (VH) results in delays in the acceptance or refusal of vaccines despite the availability of vaccination services, and it has contributed to large outbreaks of vaccine-preventable diseases in several countries, including the United States [1,2,3,4]. To prevent such outbreaks, one important strategy is maternal vaccination, which prevents diseases in mothers, neonates, and infants [5,6,7]. Despite substantial evidence for their safety, vaccines face global challenges from existing and new outbreaks during pregnancy [8]. The World Health Organization has defined VH as a refusal or delay in the acceptance of vaccines despite the availability of vaccination services. It has been acknowledged that VH is context-specific, and it depends on factors like complacency, the convenience of vaccines, and confidence in vaccines [9,10]. To improve vaccination programs, it is essential to know the barriers among stakeholders.

Vaccine acceptance and hesitancy (VH) are influenced by multiple factors that vary by time, setting, and vaccine type [11,12]. Among pregnant women, vaccination decisions are particularly complex, as they affect both maternal and fetal health [13]. Although studies have examined barriers to vaccination, few have investigated when parents make these decisions. A previous study found that 88% of 195 pregnant women made their vaccination decision during pregnancy, with a strong association (η = 0.87) between antenatal intentions and subsequent vaccination behavior [14]. Other studies reported that approximately 28% of parents were undecided about vaccination before childbirth, with a similar proportion remaining hesitant or uncertain postpartum, often resulting in delayed or refused vaccination [15,16]. Henrikson et al. observed a significant decline in maternal vaccine hesitancy from birth to 2 years and suggested that the antenatal and early postpartum periods may provide important opportunities to support informed vaccination decision-making [17]. Another study showed that training antenatal mothers improves vaccination knowledge, attitudes, and practices in both mothers and newborns [18]. These studies provide evidence that parental exploration and consolidation of vaccine attitudes occur in the antenatal period. Hence, the antenatal period provides an window of opportunity for the optimal understanding of vaccination decisions.

The United Arab Emirates’ national vaccination policy has embedded vaccination in the national agenda since 2021, and aims to achieve 100% vaccination rates for the population, including antenatal women, aligning with sustainable development goals for 2030 [19]. Based on these facts, we investigated the knowledge, attitudes, and practices of mothers attending antenatal clinics, and explored the factors contributing to VH. The questions covered all vaccines according to the national immunization schedule for children and antenatal women. The research questions posed were as follows: What are the levels of vaccination knowledge, attitudes, and practices among women in the antenatal period, and what are the factors influencing vaccination acceptance or hesitancy among antenatal women?

## 2. Materials and Methods

This is a cross-sectional study conducted at secondary care hospitals and in the community in Ras Al Khaimah, United Arab Emirates. Antenatal women attending the outpatient clinics of community health centers and Abdullah Bin Omran Hospital for Obstetrics and Gynecology were invited to participate in the survey. After being informed about the study, those who signed consent were either sent an online form or given a validated questionnaire in Arabic or English (according to their preference). Women who were not currently or recently pregnant, and those who did not give their consent, were excluded.

### 2.1. Sample Size and Sampling

An online sample size calculator available at Calculator.net was used to determine the sample size [20]. Approximately 2100 women attend the antenatal clinic of the hospital every year. At a 95% confidence level, with a 5% margin of error and a 50% population proportion, the calculated sample size was 326. A continuous purposive sampling was performed until the required number of participants was reached.

### 2.2. Data Collection Tool

The demographic data were collected. We followed the existing guideline for developing the questionnaire [21]. We prepared the initial version of the questionnaire after reviewing the relevant literature [22]. It contained 40 items (Questionnaire version 1). The questions covered all vaccines according to the national immunization schedule for children and antenatal women. We invited faculty members who are experts in mixed-methods research and community medicine (*n* = 10) from the institution to screen the questionnaire for content. We then invited 10 other participants to test the questionnaire for general readability and comprehension. The questionnaire was then modified according to their inputs to create the final questionnaire (Questionnaire version 2) containing 27 items assessing the participant’s knowledge, attitude, and practices, which was used after pilot testing. We tested the overall reliability of the items, and the Cronbach α values obtained for each question ranged between 0.77 and 0.82, suggestive of acceptable internal consistency. The validated questionnaire was used for the survey. The questionnaire included questions with options of strongly positive, positive, negative, and strongly negative. In these questions, the first two options were included as positive responses, and the latter two were considered as negative responses. The questionnaire items are included in the tables.

### 2.3. Data Analysis Plan

The data entered in Microsoft Excel were coded and entered into SPSS (version 28) (IBM Corp., Armonk, NY, USA). Descriptive statistics including means, medians, and proportions were used to present participant characteristics and demographics. Data were expressed as rates and percentages. For continuous variables, means and standard deviations were calculated. For the categorical variables, the chi-square test was used. A *p*-value < 0.05 was considered significant. Thematic analysis of responses to open questions was conducted using Braun and Clarke’s six-phase framework [23].

### 2.4. Generative AI Use

The authors used generative artificial intelligence (AI) tools ChatGPT 4.0 and Grammarly (Version 14.1153.0) during the preparation of this manuscript to support language refinement and improve clarity and readability. The authors reviewed and verified all content and take full responsibility for the manuscript’s accuracy and integrity.

## 3. Results

A total of 407 women were invited to participate in this study, of whom 352 consented, and finally 326 women were enrolled. Of those enrolled, 69.9% (228/326) were 30 years of age or older, and 39.2% (*n* = 128) were employed, although the majority were college-educated or higher (74.5%; *n* = 243). A 43.5% share of the participants were Emiratis, followed by Southeast Asians (*n* = 135). The majority of women had total household monthly income of AED 10,000 or higher (54.6%; *n* = 178) (Table 1).

There were 21 women in their first pregnancy (primigravida, 4.1%), while other women had between one and 10 children (95.8%). The mean gestational age was 28.05 ± 3.58, and 16 women had just delivered, whereas most women who agreed to participate were in the second trimester between 14 and 27 weeks (*n* = 158; 48.46%).

A 94.7% share of participants were aware of places where vaccines are available, whereas 17 (5.2%) women were unaware of the vaccine facilities. The reasons stated included being a primigravida in early pregnancy, when information may not have yet been provided routinely, or being new to the country. Sixty-four percent of the women relied on more than one information source; 0.4% trusted information received about vaccination, 89% revealed that they rely only on doctors (general physicians or OB/GYN or pediatricians) for vaccine-related information, and 7% on other healthcare professionals. Additionally, 32.5% relied on information from the media, either alone or alongside other sources. Only three individuals searched specific sites like the CDC website for information, and two women relied only on vaccinologists for vaccine-related information. This highlights the continued importance of healthcare professionals as primary vaccine educators alongside the growing influence of social media (Table 2).

Most respondents (67.4%) did not believe that new vaccines carry more risks than older vaccines, though those who did expressed concerns about lack of long-term data and unfamiliar technology. Most participants (93.5%) held a positive attitude toward vaccination as a means of protecting their children from disease, and 78.8% believed that receiving more vaccines is beneficial, while a smaller proportion felt vaccination should be limited to those only recommended or for life-threatening diseases. Notably, 42.02% of respondents personally knew someone who had experienced post-vaccination side effects, most of which were minor, such as swelling, fever, or menstrual irregularities, suggesting that personal experiences within social circles may influence vaccine perceptions.

Most respondents demonstrated strong confidence in vaccine safety, with strong positive and positive responses (*n* = 310; 95%). A favorable attitude was also seen, with 273 (83.7%) agreeing that the benefits of vaccines outweigh the risks [Table 3]. A large proportion of respondents (305 combined) believed vaccination can prevent severe illness, with no participant selecting a strongly negative response to this statement. Concerns about serious side effects were expressed by 203 respondents (62.2%), combining strong positive and positive responses. Anxiety about the side effects was still expressed by 123 participants, either strongly or moderately. Moreover, 291 respondents were confident that following the recommended vaccination schedule is beneficial for their child, and the majority (*n* = 219) disagreed with the notion that natural illness builds immunity better than vaccination. However, 159 (48.7%) self-reported vaccine hesitancy, although only four participants had strong hesitancy (Table 3).

Among the 326 respondents with pregnancy history, 165 received vaccines during previous pregnancies, while 140 did not, with the most common reason being prior full vaccination before pregnancy (98 cases), followed by uncertainty about whether an injection was vaccine-related (53 cases) and lack of information about vaccination options (17 cases) (Table 4). Most participants (*n* = 257) intend to receive all recommended vaccinations in future pregnancies, though 69 do not, with the most common reason being that they are not planning additional pregnancies (47 cases). Regarding child vaccination practices, 320 of 326 respondents would vaccinate their children according to recommended schedules, with only about 2% (*n* = 6) not doing so, reflecting a very high intention to adhere to vaccination schedules when they do vaccinate. Of the 326 respondents, 39 reported deciding not to vaccinate their child for reasons other than illness or allergy, citing unavailability and geopolitical reasons.

For COVID-19 vaccination, 224 (68.7%) respondents vaccinated their child; the most common reason for not vaccinating was that the child was younger than the recommended age (73 cases). About 95% of women (*n* = 308) reported that they generally follow their doctor or healthcare provider’s recommendations about vaccination for their children.

Bivariate analysis was performed to compare appropriate responses for knowledge, attitude, and practice questions between groups. Scores of 0 and 1 were assigned for incorrect and correct responses, respectively. Where applicable, groups were combined to represent two comparators. Comparisons show that education beyond school, household income of more than AED 10,000, and inadequate knowledge and attitude were significantly associated with self-reported acceptance or hesitancy (Table 5). The strongest correlation for acceptance was having an appropriate attitude toward vaccination. It encompassed education, income, and even knowledge. Vaccine hesitancy was self-reported by 159 participants (48.7%), although only four had strong hesitancy. This was applicable both to them and their present and future children.

The logistic regression test shows education, household income, knowledge, and attitude as significant predictors of vaccine acceptance/hesitancy (Table 6).

As VH includes complacency, convenience, and confidence, open-ended questions were asked to explore specific views covering these aspects. Those who had strong VH expressed mistrust, social media information, and religious beliefs as influencing factors. Most of the participants expressed their views on COVID-19 vaccines because the pandemic was recent. However, many women could recall other vaccines they or their children received. A thematic analysis of the statements was conducted, and themes with the verbatim quotes are presented in Table 7.

Thematic analysis of the reasons for hesitancy revealed concerns about the content of the vaccine as the most common association with subthemes, as well as societal pressure against the content and contradictory beliefs. Among the participants, 3.2% (*n* = 10) were hesitant due to beliefs that vaccines are not effective in preventing the disease or have serious side effects. Although hesitancy is positively correlated with being a homemaker and parity, the difference was not statistically significant. It is important to interpret the themes accurately. While misinformation, lack of adequate knowledge, and societal pressures can cause VH, which can be mitigated through creating awareness, managing refusal for vaccination despite adequate knowledge would require a deeper understanding of sociocultural issues.

## 4. Discussion

This study provides a comprehensive examination of vaccine knowledge, attitudes, and practices among pregnant women in the United Arab Emirates, revealing both encouraging trends and persistent challenges in maternal and childhood vaccination acceptance. The findings contribute to the growing body of literature on vaccine hesitancy and offer insights for targeted interventions in this population.

The study demonstrated generally high levels of vaccine awareness, with 94.7% of participants knowing where to access vaccinations and 94.17% knowing where to obtain reliable vaccine information. This is consistent with previous research indicating that healthcare providers remain the primary source of vaccine information for pregnant women [2]. Notably, 89% of respondents relied exclusively on doctors for vaccine-related information, underscoring the critical role of healthcare professionals as trusted messengers [1,6]. This finding aligns with research by Wilson et al. [8], who identified healthcare provider recommendation as one of the strongest predictors of vaccine acceptance during pregnancy. However, the growing influence of media as an information source (utilized by 32.5% of participants) warrants attention. This dual reliance on professional and media sources creates potential for conflicting information that may contribute to hesitancy [12,24]. Only three participants sought information from official sources like the CDC website, suggesting opportunities to improve digital health literacy and promote evidence-based resources [25].

Most participants showed positive attitudes toward vaccination, with 93.5% believing vaccines protect children from disease and 83.7% agreeing that benefits outweigh risks. These findings are encouraging compared to studies in other contexts where vaccine confidence has eroded significantly [26,27]. Vaccine acceptance is contextual, and several factors shape VH. A recent study among oncology patients identified appropriate communication as key to vaccine acceptance [28]. Nearly 95% of participants in our study were aware of the vaccine services. This reflects the effectiveness of communication during antenatal care. The high confidence in vaccine safety (95% expressing strong or positive confidence) may reflect effective public health communication in the UAE context [18]. Nevertheless, the study identified important information on vaccine attitudes. While 78.8% believed more vaccines are beneficial, a substantial minority (46.31%) questioned the need for vaccines against uncommon diseases. This reflects a common theme in the vaccine hesitancy literature—the perceived irrelevance of diseases that have become rare due to vaccination success [3,11]. Similarly, 73.9% disagreed that vaccination is only for daycare/school entry, suggesting most understand vaccination’s broader health benefits.

The finding that 42.02% personally knew someone with post-vaccination side effects (mostly minor) is significant. Personal experiences within social networks can influence vaccine perceptions, sometimes more than available medical evidence [14,16]. This aspect of vaccine experiences aligns with research on the role of narratives in vaccine decision-making [29]. Similarly, the self-reported hesitancy rate of 48.7% appears surprisingly high given the generally positive attitudes expressed. This discrepancy requires careful interpretation. It may reflect participants’ individualistic understanding, i.e., acknowledging some hesitation while maintaining overall favorable vaccination intentions [9]. The finding that only four participants reported strong hesitancy supports this interpretation, suggesting that for most, hesitancy is conditional rather than absolute [30,31]. Practice patterns revealed high vaccination intention, with 97.5% (320/326) vaccinating children according to schedules and 95% following provider recommendations. This intention–behavior alignment is crucial, as provider recommendation is among the strongest determinants of vaccine uptake [13,32]. A study of COVID-19 vaccine refusal among patients with lung cancer also highlighted the fact that safety concerns and payment for the vaccine were significant factors for opting against vaccination [33]. The high acceptance of COVID-19 vaccination for children (68.7%) is notable, given documented hesitancy towards this vaccine in other populations [34,35].

The thematic qualitative analysis revealed six key themes explaining vaccine hesitancy, which align with established determinants in the WHO’s Behavioral and Social Drivers (BeSD) framework [36,37]. Safety and risk concerns dominated, with participants expressing fears about new vaccine technology, lack of long-term data, and potential effects on the baby. These concerns reflect broader societal debates about vaccine safety and the challenge of communicating about emerging technologies [5,7]. In its qualitative insights, a recent systematic review on VH among healthcare workers in Europe reported similar findings of concerns regarding vaccine safety and information gaps as factors affecting VH [38]. Distrust and skepticism emerged as another significant theme, with participants expressing distrust of pharmaceutical motives and conditional trust dependent on mandates. This resonates with the recent literature on political and commercial distrust as drivers of hesitancy [26,30,39]. The conditional nature of trust (“only if mandatory”) suggests that while participants may follow requirements, they do not necessarily embrace vaccination voluntarily [4]. Knowledge gaps were evident, with participants reporting a lack of information and reliance on informal sources. Other influences, such as religious beliefs, also shaped VH in our study, aligning with previous reports [40]. This highlights the importance of clear, consistent communication from healthcare providers throughout pregnancy and early childhood [17,41,42]. The finding that some participants were unsure whether injections received during pregnancy were vaccines suggests opportunities to improve patient–provider communication about antenatal vaccinations.

### 4.1. Comparison of Quantitative and Qualitative Findings

The convergent parallel mixed-methods approach reveals important complementarity between quantitative and qualitative findings. While quantitative data showed generally positive attitudes and high vaccination intentions, qualitative responses exposed underlying concerns and conditional acceptance that quantitative scales may not fully capture. For example, although 93.5% agreed vaccines protect against disease, qualitative responses revealed that many viewed vaccines as only necessary for severe diseases or as compliance requirements. Similarly, the discrepancy between high vaccine confidence (95%) and moderate hesitancy rates (48.7%) is better understood through qualitative insights, where participants expressed both general support for vaccination and specific contextual concerns. This finding supports the argument that hesitancy is not a binary state but exists on a continuum, influenced by multiple factors and amenable to tailored interventions [9,30,43].

The findings align with international research on maternal vaccination attitudes. In accordance with other studies, this study found that safety concerns and limited long-term data were significant barriers [5,7]. The reliance on healthcare professionals as information sources aligns with findings among midwives, who identified healthcare providers as key vaccine educators [41]. The role of social media and informal sources in shaping vaccine attitudes is well documented [25,27,34]. This study’s finding that over a third of participants used media sources for vaccine information, combined with the thematic evidence of informal information-seeking, supports calls for improved digital health communication strategies [44,45].

Lower educational attainment and family income may contribute to vaccine hesitancy through interconnected pathways involving access to health information, vaccine knowledge, trust, and attitudes. Limited knowledge or difficulty evaluating health information may increase susceptibility to misconceptions, while negative vaccine attitudes can further shape the information individuals seek and trust. Vaccine hesitancy is therefore influenced by multiple interacting individual, social, and contextual factors rather than a single determinant [30]. Social media may reinforce these vulnerabilities by rapidly disseminating misinformation. In a randomized study, exposure to online vaccine misinformation significantly reduced vaccination intention in both the UK and USA, with effects varying across sociodemographic groups [46]. Family, community, and broader societal norms may further reinforce these influences, particularly where trust in science and health authorities is low. Evidence from 126 countries indicates that societal trust in science is associated with greater vaccine confidence, beyond individual-level trust [47]. Thus, socioeconomic disadvantage, limited knowledge, negative attitudes, online misinformation, and social influences may interact to reinforce vaccine hesitancy.

These findings have several practical implications. First, the high trust in healthcare providers suggests that training programs for obstetricians, midwives, antenatal mothers, and general practitioners on vaccine communication could significantly impact vaccine acceptance, as seen with other conditions [32,48,49,50,51,52]. Second, the identified knowledge gaps indicate the need for clear, consistent, and repeated education about vaccines during antenatal care, addressing concerns about new vaccine technology, long-term safety, and specific side-effect profiles [1,19]. Third, misinformation and reliance on informal sources call for stronger health communication strategies that leverage trusted community channels [44,53]. Community engagement approaches, particularly with minority populations, may help address culturally specific concerns [53,54]. Fourth, the finding that logistical barriers and vaccine availability were mentioned by some participants suggests addressing access issues could improve uptake [15,24]. As suggested by recent research on effective communication regarding vaccines, the gaps identified included distinguishing between vaccine hesitancy and vaccine refusal, and the need for research that reflects greater geographic, cultural, and socioeconomic diversity, among others [55]. Hence, this research has the potential to contribute to better understanding of VH.

### 4.2. Strengths and Limitations

This study has several limitations. The cross-sectional design cannot establish causal relationships between VH determinants and vaccination behavior. The study was conducted at a single Emirate in the UAE, affecting generalizability to other regions or populations. Self-reported data may be subject to social desirability bias, particularly regarding vaccination attitudes. Furthermore, the exploratory nature of the qualitative components prevented a comprehensive analysis of VH components such as convenience, confidence, and complacency. Although the questions covered all vaccines in the national immunization schedule for children and antenatal women, the timing of data collection relative to the COVID-19 pandemic may have influenced vaccine attitudes and recall. Additionally, the study did not capture detailed information on specific vaccines, which may mask important differences in attitudes toward different vaccines. The low proportion of primigravida women (4.1%) limited exploration of first-time mothers’ unique concerns, despite evidence suggesting parity influences vaccine acceptance.

However, this study also demonstrates several strengths. The mixed-methods design provides a comprehensive understanding of both quantitative patterns and qualitative nuances of vaccine hesitancy. The large sample size (*n* = 326) enhances generalizability within the UAE context. The focus on the antenatal period is timely given the increasing emphasis on maternal immunization, and including both Emirati and expatriate participants provides multicultural perspectives.

## 5. Conclusions

This study reveals a population with generally favorable vaccine attitudes and high vaccination intentions, alongside significant underlying hesitancy characterized by safety concerns, knowledge gaps, and conditional trust. Healthcare professionals remain the most trusted information source, and efforts to strengthen vaccine communication during antenatal care could further improve acceptance. Addressing the identified barriers through evidence-based, culturally sensitive, and community-engaged strategies will be essential to maintain high vaccination coverage and prevent future outbreaks.

## Figures and Tables

**Table 1:** Participant characteristics.

Characteristics	Total *n* = 326			
Age	Mean and SD 33.58 ± 2. 87	Age ≤ 30 years 30% *(n* = 98)	Age > 30 years 69.9% (*n* = 228)	
Nationality	Emirati 43.5% (*n* = 142)	Southeast Asian 41.4% (*n* = 135)	Arab (other nationalities) 15.1% (*n* = 49)	
Education	Junior school and lower 3.06% (*n* = 10)	Senior school 22.3% (*n* = 73)	College and above 74.5% (*n* = 243)	
Employment	Homemaker 60.7% (*n* = 198)	Employed 39.2% (*n* = 128)		
Monthly household income	<5000 AED 17.4% (*n* = 57)	5000–10,000 AED 27.9% (*n* = 91)	>10,000 AED 54.6% (*n* = 178)	
Parity	Mean = 2.58 (±1.2) Range (0–10)	Primigravida 4.1% (*n* = 21)	Parity ≥ 1, 95.8% (*n* = 305)	
Gestational age		1st trimester 8.89% (*n* = 29)	2nd trimester 48.46% (*n* = 158)	3rd trimester 37.7% (*n* = 123)

**Table 2:** Knowledge about vaccination.

Parameter	Responses *n* (%)		Reason Stated
	Yes	No	
Q1. Do you know where you can get your vaccinations?	309 (94.7%)	17 (5.2%)	- Were informed in the antenatal period by doctor or nurse - Were informed by my child’s pediatrician
Q2. Do you know where you can get reliable information about vaccinations?	307 (94.17%)	19 (5.8%)	
Q3. Did you ever receive information about vaccination during pregnancy?	317 (97.2%)	09 (2.7%)	
Q4. I trust the information received about vaccines.	295 (90.4%)	31 (9.5%)	I trust reliable sources only; I also search from other sources; Some differences between the sources of information
Q5. New vaccines carry more risks than older vaccines.	106 (32.5%)	220 (67.4%)	Lack of information, not enough studies, no long-term data, I heard that they are risky, it is a new technology, not sure, The world has become too greedy, read somewhere
Q6. The only reason to have childhood vaccination is that they can start daycare or school.	85 (26.07%)	241 (73.9%)	It is a mandatory requirement
Q7. Do you know anyone who has had complications or side effects after taking any vaccine?	137 (42.02%)	189 (57.9%)	Minor ones, swelling and itching at site, loss of smell, menstrual problems, fever
Q8. I believe getting vaccines is a good way to protect my child/children from disease.	305 (93.5%)	16 (4.9%)	No belief in vaccination
Q9. I believe that the more vaccinations children receive, the better it is for them.	257 (78.8%)	69 (21.1%)	Unnecessary/good, only recommended, only for life-threatening diseases, individualized
Q10. I believe my child/children doesn’t/don’t need vaccines for diseases that are uncommon nowadays.	151 (46.31%)	176 (53.98%)	If not required, can cause harm
Q11.* Which is/are your reliable source of information?			
General physician practitioner Obstetrician (OBGYN doctor) Pediatric doctor Family/friends Social media Others (healthcare professional, newspaper, Journal, websites like CDC, Google search, vaccine specialist)	169 154 101 83 106 77	51.8% 47.2% 30.9% 25.4% 32.5% 23.6%	

* The numbers do not add up to *n*, as the responses were not exclusive and included multiple sources.

**Table 3:** Attitudes about vaccination.

Statements	Strong Positive; *n* (%)	Positive; *n* (%)	Negative; *n* (%)	Strong Negative; *n* (%)
Q12. How confident are you in the safety of vaccines?	83 (25.4%)	227 (69.6%)	13 (3.9%)	3 (1%)
Q13. Do you agree that the benefits of vaccines outweigh the risks?	109 (33.4%)	164 (50.3%)	50 (15.3%)	3 (1%)
Q14. I believe that many of the severe illnesses can be prevented by vaccination.	127 (38.9%)	178 (54.6%)	21 (6.4%)	0
Q15. How concerned are you about the potentially serious side effects following vaccination?	12 (3.6%)	191 (58.5%)	67 (20.55%)	56 (17.17%)
Q16. How convinced are you about the vaccine efficacy?	49 (15%)	173 (53.06%)	98 (30%)	6 (1.89%)
Q17. How sure are you that following the recommended schedule is good for your child?	138 (42.33%)	153 (46.9%)	35 (10.7%)	0
Q18. Do you agree that your child’s immunity develops when he or she gets sick more than when he or she gets vaccinated?	27 (8.28%)	80 (24.5%)	186 (57%)	33 (10.1%)
Q19. Overall, how hesitant do you consider yourself to be about vaccinations?	4 (1.22%)	155 (47.5%)	122 (37.4%)	45 (13.8%)

**Table 4:** Practice of vaccination.

Questions	Number	If No, Why (Number of Responses)
Q20. Did you get any vaccine in your previous pregnancy?	Yes = 165 No = 140 NA = 21 (Primigravida)	Do not remember; it was long time ago (18, 21, 24 years ago) (*n* = 3) Not sure whether the injection received in pregnancy was a vaccine (*n* = 53) There were no vaccines available at the time (*n* = 11) I was not informed of any vaccination (*n* = 17) It was not necessary (*n* = 4) I was fully vaccinated before pregnancy, and it was not required (*n* = 98) I was worried about the future effect of vaccination on the baby *(n* = 18)
Q21. Do you intend to get all the recommended vaccinations in your future pregnancies?	Yes = 257 No = 69	Not planning any more pregnancies (*n* = 47) Will decide at that time (*n* = 15)
Q22. Do you vaccinate your child according to recommended schedules?	Yes = 320 No = 6	I do not trust vaccination (*n* = 1) Schedules can be adjusted if missed (*n* = 1)
Q23. Did you ever decide on not vaccinating your child for reasons other than illness or allergy?	Yes = 39 No = 288	Unavailability of some vaccines in my own country (*n* = 2)
Q24. Did you take COVID-19 vaccine?	Yes = 207 No = 119	My health condition does not allow me to receive vaccine (*n* = 14) Will receive shortly (*n* = 65) Others (*n* = 40)
Q25. Did you give your child the COVID-19 vaccine?	Yes = 224 No = 102	Younger than recommended age (*n* = 73) Planning to give soon (*n* = 21) Scared/worried of/about vaccines (*n* = 3) Fear of side effects (*n* = 5)
Q26. I will give the vaccine to my child if other children are vaccinated.	Yes = 263 No = 63	Only if it is mandatory to give that vaccine I do not follow others
Q27. Generally, I do what my doctor or health care provider recommends about vaccines for my child/children.	Yes = 308 No = 18	I also search from other sources (*n* = 5)

**Table 5:** Factors associated with vaccine acceptance/hesitancy.

Characteristics	Acceptance *n* (%); 167 (51.3%)	Vaccine Hesitancy *n* (%);159 (48.7%)	*p*-Value
Age < 30 (*n* = 98) Age > 30 (*n* = 228)	51 (52%) 116 (50.9%)	47 (47.9%) 112 (49.1%)	0.847
Up to senior school (*n* = 83) College and above (*n* = 243)	36 (43.3%) 141 (58%)	47 (56.6%) 102 (41.9%)	0.020
Homemaker (*n* = 198) Employed (*n* = 128)	101 (51.1%) 66 (51.6%)	97 (48.9%) 62 (48.4%)	0.922
Income up to AED 10,000 (*n* = 148) Income > AED 10,000 (*n* = 178)	63 (42.5%) 104 (58.5%)	85 (57.4%) 74 (41.5%)	0.004
Primigravida (*n* = 21) Parity ≥ 1 (*n* = 305)	9 (42.8%) 158 (51.8%)	12 (57.1%) 147 (48.1%)	0.427
Adequate knowledge (*n* = 297) Inadequate knowledge (*n* = 29)	159 (53.5%) 08 (38%)	138 (46.46%) 21 (72.4%)	0.007
Appropriate attitude (*n* = 288) Inappropriate attitude (*n* = 38)	161 (55.9%) 06 (15.7%)	127 (44%) 32 (84.2%)	<0.0001

**Table 6:** Logistic regression of vaccine hesitancy (*n* = 159/326).

Variable	Category	Hesitant/Total	Crude OR (95% CI)	Crude *p*	Adjusted OR (95% CI) *	Adjusted *p* *
Age	<30	47/98	0.96 (0.59–1.55)	0.85	1.10 (0.63–1.92)	0.73
	≥30	112/228	1.00 (ref)		1.00 (ref)	
Education	Up to senior school	47/83	1.80 (1.09–2.99)	0.020	2.05 (1.15–3.65)	0.014
	College and above	102/243	1.00 (ref)		1.00 (ref)	
Occupation	Homemaker	97/198	1.02 (0.65–1.59)	0.92	1.24 (0.73–2.09)	0.43
	Employed	62/128	1.00 (ref)		1.00 (ref)	
Income	≤10,000 AED	85/148	1.90 (1.22–2.95)	0.004	1.85 (1.11–3.06)	0.018
	>10,000 AED	74/178	1.00 (ref)		1.00 (ref)	
Parity	Primigravida	12/21	1.43 (0.59–3.51)	0.43	1.62 (0.59–4.42)	0.35
	Parity ≥1	147/305	1.00 (ref)		1.00 (ref)	
Knowledge	Inadequate	21/29	3.02 (1.31–6.98)	0.007	2.71 (1.10–6.66)	0.030
	Adequate	138/297	1.00 (ref)		1.00 (ref)	
Attitude	Inappropriate	32/38	13.9 (4.2–46.0)	<0.0001	12.6 (3.7–42.4)	<0.0001
	Appropriate	127/288	1.00 (ref)		1.00 (ref)	

*Adjusted model: all seven variables entered simultaneously (binary logistic regression, hesitancy = 1). Hosmer–Lemeshow *p* ≈ 0.63; model χ^2^(7) large, *p* < 0.001.

**Table 7:** Vaccine hesitancy and factors affecting appropriate knowledge and attitude.

Theme [VH Component]	Subtheme	Verbatims
1. Safety and Risk Concerns [Confidence]	Fear of side effects	“I heard that they are risky”; “Fear of side effects”; “Scared/worried of vaccines”; “Minor ones, swelling and itching at site, loss of smell, menstrual problems, fever”
	Concerns about new vaccine technology	“It is a new technology”; “Lack of information, not enough studies, no long-term data”; “New vaccines carry more risks than older vaccines”
	Concerns about effect on baby	“I was worried about the future effect of vaccination on baby”
2. Distrust and Skepticism [Confidence + complacency]	Distrust of vaccination in general	“I do not trust vaccination”; “No belief on vaccination”
	Distrust of pharmaceutical/commercial motives	“World has become too greedy”
	Conditional trust	“I will give the vaccine to my child only if it is mandatory”; “I do not follow others”; “Will decide at that time”
3. Knowledge Gaps and Misinformation [Confidence]	Lack of awareness or information	“I was not informed of any vaccination”; “Not sure whether injection received in pregnancy was any vaccine”
	Reliance on informal sources	“Read somewhere”; “I heard that they are risky”; “I also search from other sources”
4. Perceived Necessity of Vaccines [Confidence, complacency]	Vaccines seen as only a compliance requirement	“The only reason to have childhood vaccination is they can start daycare or school”; “It is a mandatory requirement”
	Vaccines seen as unnecessary for uncommon diseases	“My child doesn’t need vaccines for diseases that are uncommon nowadays”; “If not required, can cause harm”
	Preference for selective vaccination	“Only recommended vaccines”; “Only for life-threatening diseases”; “Individualized”; “Unnecessary/only good for some”
5. Belief in Natural Immunity [Confidence *]	Natural illness preferred over vaccination	“My child’s immunity develops when he gets sick more than when he gets vaccinated”
6. Logistical, Societal, and Religious Barriers [Confidence * + convenience]	Vaccine unavailability	“Unavailability of some vaccines at my own country”
	Not allowed in religion	“It is not a natural phenomenon; hence I don’t take vaccine, nor family members”.
	Flexible attitude toward schedule adherence	“Schedules can be adjusted if missed”

* The confidence expressed in the statements against vaccination.

## Data Availability

All data generated by this research are part of this manuscript.
